# Integrating and evaluating sex and gender in health research

**DOI:** 10.1186/s12961-016-0147-7

**Published:** 2016-10-10

**Authors:** Suzanne Day, Robin Mason, Stephanie Lagosky, Paula A. Rochon

**Affiliations:** 1 Women’s Xchange, Women’s College Research Institute, Women’s College Hospital, 76 Grenville Street, Toronto, Ontario M5S 1B2 Canada; 2 Dalla Lana School of Public Health and Department of Psychiatry, University of Toronto, 27 King’s College Circle, Toronto, Ontario M5S 1A1 Canada; 3 Department of Medicine, University of Toronto, 27 King’s College Circle, Toronto, M5S 1A1 Ontario Canada

**Keywords:** Sex and gender, Health research, Equity, Research design, Reporting, Quality

## Abstract

Both sex (biological factors) and gender (socio-cultural factors) shape health. To produce the best possible health research evidence, it is essential to integrate sex and gender considerations throughout the research process. Despite growing recognition of the importance of these factors, progress towards sex and gender integration as standard practice has been both slow and uneven in health research. In this commentary, we examine the challenges of integrating sex and gender from the research perspective, as well as strategies that can be used by researchers, funders and journal editors to address these challenges. Barriers to the integration of sex and gender in health research include problems with inconsistent terminology, difficulties in applying the concepts of sex and gender, failure to recognise the impact of sex and gender, and challenges with data collection and datasets. We analyse these barriers as strategic points of intervention for improving the integration of sex and gender at all stages of the research process. To assess the relative success of these strategies in any given study, researchers, funders and journal editors would benefit from a tool to evaluate the quality of sex and gender integration in order to establish benchmarks in research excellence. These assessment tools are needed now amidst growing institutional recognition that both sex and gender are necessary elements for advancing the quality and utility of health research evidence.

## Sex and gender in health research: a persisting data gap

To inform the development of effective and equitable healthcare policies, programs and delivery models, health researchers have long been called upon to examine the impact of sex and gender on health outcomes, health-related behaviours and service provision [1–4]. A number of international funding mechanisms have developed policies requiring sex and gender to be integrated in research proposals, including WHO, The European Commission Directorate-General for Research and Innovation, the Canadian Institutes of Health Research (CIHR), and the United States National Institutes of Health [5]. The European Association of Scientific Editors has formed a Gender Policy Committee to improve sex and gender reporting practices across all scientific fields [6]. Some peer-reviewed journals have also developed sex and gender reporting policies [7]. Building on this momentum, a global call to action has been issued for gender to be included in the research impact assessment undertaken by research funders, institutions and evaluators in order to inform more equitable health policy and practice [8].

Despite this widespread and growing recognition that including sex and gender is integral to better evidence, the need for better integration has been acknowledged across a range of health and biomedical fields [9–18]. A recent study of research designs in funding applications to CIHR found that uptake of sex and gender is uneven in health research and varies by discipline: sex was most often incorporated in clinical research, gender most often in population health research, and both sex and gender were least often incorporated in biomedical studies [9]. Additionally, reporting on sex and gender in health research publications has yet to be adopted as standard practice [10, 19]. A review of sex and gender in medical literature found that, while incorporation of these factors in study design and evaluation has increased over time, the total number of publications remains small and underrepresentation of sex and gender differences is widespread [20]. Similarly, a review of sex and gender in randomised controlled trials in high-impact international journals found that only 6% of the reviewed papers that included both men and women examined interactions between sex or gender and other variables of interest in the study [21]. Clearly, challenges persist.

As one of 12 research centres comprising the Ontario Strategy for Patient-Oriented Research Support for People and Patient-Oriented Research and Trials Unit, Women’s Xchange works with health research teams to improve the quality of sex and gender integration throughout all stages of their research projects. In this commentary, we expand upon recent observations that a tendency towards ‘path dependency’ has hindered progress in considering sex and gender in health research systems [8], offering some insights into the difficulties of changing the status quo in health knowledge production. Drawing on our experiences in consulting on health research projects, we identify and examine the persisting challenges with sex and gender integration from the researcher perspective. We discuss some of the strategies that have been proposed by sex and gender scholars to address these challenges, concluding with a call to evaluate how well these variables have been integrated in health research.

## The challenges in (and strategies for) sex and gender integration

From the researcher perspective, we have identified four interrelated challenges to incorporating sex and gender in health research, which can be summarised as follows: the use of inconsistent terminology, difficulties in applying the concepts, a failure to recognise the impact of sex and gender on research design and outcomes, and challenges with data collection and datasets. Each will be explored below, along with potential strategies for moving forward.

### Inconsistent terminology

First, sex and gender are often used interchangeably and incorrectly in scientific writing, causing confusion and obscuring the relationships and insights that would otherwise be revealed [22, 23]. General acceptance and use of agreed upon definitions, such as those endorsed by CIHR (Table 1), may go some way in resolving this confusion [24].

**Table 1:** Canadian Institutes of Health Research (CIHR) definitions of sex and gender [24]

Variable	Definition
Sex	The biological and physiological characteristics that distinguish males from females^a^
Gender	The socially constructed roles, expectations, relationships, behaviours, relative power, and other traits that societies ascribe to women, men and people of diverse gender identities^b^

^a^It should, however, be recognised that the assumption that the population can be divided neatly into male/female categories does not accurately reflect the full diversity of human biology, given that approximately 1.7% of the population is intersex [45]; hence the concept of sex as a male/female binary is, itself, a social process [46]

^b^There is a need in health research to move beyond static, binary categorisations of gender, given that gender exists on a continuum and is dynamic in varying over time and across cultures [36]. Researchers are cautioned to examine their tools and analyses for potentially reproducing the gender binary and rendering invisible the experiences of persons for whom binary categorisations may be insufficient, including transgendered persons [47]

While these definitions may appear to categorise sex and gender as mutually exclusive concepts, in reality, sex and gender are interrelated and intersect. The body is at once both biological and social [25], and thus physical health is simultaneously influenced by both sex (the biological) and gender (the social). For example, both biological processes as well as factors shaped by gender (including clothing, physical activity and occupation) are implicated in bone health [25]. Similarly, differences in both sex (e.g. gene expression, protein patterns) and gender (e.g. patients’ health seeking behaviour, physicians’ disease management strategies) are factors in the manifestation and outcomes of heart disease [26]. Agreement on definitions of sex and gender that can be used across the fields of health research – as well as accurate use of each term – is an important first step in working through these complexities [27].

### Applying the concepts

Applying the concepts of sex and gender represents another challenge for health researchers. Examples of how sex and gender can influence the spectrum of health and disease include gene expression, hormone levels and enzymatic processes (biological: sex) and differences in societal roles, societal perceptions and factors affecting healthcare access (social: gender) [14]. Capturing these variables requires asking different research questions, designing and taking up different kinds of research, and identifying and observing different phenomena. The meaningful integration of sex and gender requires attending to these factors from the outset. Thinking about sex and gender will inform the formation of and approach to research questions and the literature review, shaping the orientation of the research through to analysis, interpretation, reporting and recommendations [12]. Proactively considering sex and gender from the beginning thus helps to avoid the costly, inefficient and insufficient revisiting of research to ‘add in’ sex and gender post-hoc [28].

Part of the challenge in applying the concepts of sex and gender is with determining the explanatory limitations of these concepts. For instance, disaggregating data by sex may illuminate differences between males and females but will not allow exploration of the gender-based inequities in social and economic power that additionally shape health [13]. Furthermore, finding that there are sex differences in a study outcome does not necessarily tell us anything about the biological mechanisms behind this difference. To address this challenge, it is important to ensure that mechanisms be directly tested wherever possible [29]. For example, attributing occupational injuries to assumed sex differences without investigating the more precise explanatory power of differences in body size can impact the design and implementation of safety measures [30]. Misidentifying the influential mechanism thus has consequences not only for how sex and gender differences are explained, but also for developing preventative strategies.

To further challenge the application of sex and gender in research, intersectionality has emerged as an important, additional consideration. Intersectionality describes the recognition that sex and gender intersect simultaneously with other factors that shape health outcomes, including age, race and ability, among others, all from within specific historical, geographic and cultural contexts [31, 32]. In this broader and more nuanced view, sex and gender are embedded within and inseparable from a multitude of factors that influence health and wellbeing [4]. More sophisticated analyses will require health researchers to consider the ways that sex and gender interact with these additional variables in order to develop more effective and equitable policy and practice.

### Recognising the impact

As illustrated by the uneven uptake of sex and gender considerations across the health disciplines [9], challenges also remain with acknowledging and understanding the impact that these concepts can have on the evidence produced by health research. This is particularly the case in basic experimental biomedical research where sex and gender considerations may be less readily perceived as relevant in working with non-human study populations such as cells or laboratory animals [15]. There is also a tendency for sex and gender to be treated as an ‘extra’ or optional consideration and thus not necessarily incorporated as a standard research practice [12] – or only incorporated in a tokenistic way in order to satisfy funding application requirements [9]. In order to shift perspectives and practice in a meaningful way, one strategy is to emphasise that sex and gender integration leads to better health through novel research and medical discovery. Failure to consider these concepts is to risk significant sources of error, endangering patient safety [33]. Sex and gender are also crucial for developing personalised medical therapies [34], a particularly salient contribution amidst growing emphasis on patient-centred care. This requires attending not only to sex/gender differences, but also sex/gender-specific conditions [35].

### Data collection and datasets

Finally, health researchers may face challenges with obtaining the relevant data for analysing by sex and gender. This is particularly the case for health research that relies on large administrative datasets. While administrative data may often (though not always) contain basic sex disaggregated data, indicators pertinent to gender analysis (e.g. income, household composition, caregiving responsibilities) are rarely collected [13]. Researchers cannot be expected to take up a more nuanced examination of sex and gender when data collection tools do not adequately incorporate these concepts. An important strategy for moving forward will be to consider how datasets can be expanded to collect information beyond clinical indicators with limited explanatory power, as well as alternative methods and mixed-methods analyses to obtain different kinds of data. This approach is particularly salient given that attending to the influence of gender requires asking multiple and multifaceted questions, as there is no one standard way to measure gender [17].

## Next steps: evaluating sex and gender integration

To navigate the challenges assessed above, health researchers require guidance in the comprehensive and meaningful integration of sex and gender. Many attempts have been made to provide such resources, including case studies illustrating the benefits of including sex and gender in health and medicine [2, 12, 28, 36], guidelines for integrating sex and gender in basic experimental biomedical designs [15], tools for implementing analysis of sex and gender in systematic reviews [37], curriculum development for sex- and gender-specific emergency medicine [38], recommendations for including gender in research impact assessment [8], and good practice guidelines for investigating sex and gender differences in health [29]. To facilitate uptake of these strategies, attempts have been made to develop step-by-step questionnaires and checklists on sex and gender inclusion to guide researchers throughout each stage of the research process [39–42] (Table 2).

**Table 2:** Web-based resources for sex and gender inclusion in health research

Resource	Description
The Gender Awakening Tool [39]	Provides a checklist to determine whether relevant sex and gender considerations have been included in life sciences research
Gendered Innovations [40]	Provides methods for integrating sex and gender into each stage of the research process, from design to knowledge translation
Sex and Gender in Systematic Reviews: Planning Tool [41]	Provides a step-by-step guide for asking questions about sex and gender when planning a systematic review
Toolkit Gender in EU Funded Research [42]	Provides guidance in conducting gender-sensitive scientific research

There is additionally a need to carefully consider the quality of sex and gender integration in health research and whether the uptake of these concepts is occurring in a meaningful way (when it happens at all). While existing guidelines and checklists are useful starting points for thinking through the inclusion and impact of sex and gender, to date, we are unaware of any accompanying evaluation system to assess the relative success of this integration throughout a given project. In other words, there is currently a lack of standards by which to assess whether and to what degree researchers are rising to the challenges of sex and gender integration. One notable attempt to reconcile this gap is offered by Tomas et al. [43], who have developed a questionnaire that allows reviewers to distinguish between three levels in assessing the strength of sex/gender integration in a project. However, this tool is limited to yes/no answers, namely whether sex/gender has been considered at a given stage in the project or not, which does not allow either reviewers or researchers to evaluate the relative quality of sex/gender integration throughout the project.

We propose that a scaled evaluation strategy will help to address calls to incorporate attention to sex and gender in the peer review decision-making process [10], giving reviewers a standardised protocol for assessing how sex and gender impact the rigor of a study. A scaled evaluation will also help to clarify what constitutes the ‘best practices’ for integrating sex and gender, addressing recent calls for researchers to be connected with such resources to foster routine uptake of sex and gender considerations [19]. To address these needs, and as a part of our mandate to promote sex and gender as ‘the key to better research’, our team at Women’s Xchange is presently engaged in developing and validating an assessment scale for the integration of sex and gender, applicable to both researchers and reviewers.

## Conclusions: sex and gender for stronger evidence and improved health

There is a clear need to strengthen our collective efforts to promote the integration of sex and gender as standard practice in health research. Examining the influence of sex and gender on health is an essential strategy to promote innovative, useful health research. While a number of resources have been developed towards this purpose, researchers and reviewers require more comprehensive strategies for both integration and evaluation if we are to advance the uptake of sex and gender in health research in support of better evidence. This objective has far-reaching implications for shaping decision-making at the policy and planning level. Consider, for example, the recent directive publically issued to the Minister of the Status of Women by Canada’s new federal government shortly after being elected in October 2015, calling for attention to gender in decision-making. Advancing the integration of sex and gender in health research will provide decision-makers with the necessary evidence “*to ensure government policy, legislation, and regulations are sensitive to the different impacts that decisions can have on men and women*” [44].
